# Peri-Implantitis: A Clinical Update on Prevalence and Surgical Treatment Outcomes

**DOI:** 10.3390/jcm10051107

**Published:** 2021-03-06

**Authors:** Andrea Roccuzzo, Alexandra Stähli, Alberto Monje, Anton Sculean, Giovanni E. Salvi

**Affiliations:** 1 Department of Periodontology, School of Dental Medicine, University of Bern, Freiburgstrasse 7, 3010 Bern, Switzerland; andrea.roccuzzo@zmk.unibe.ch (A.R.); anton.sculean@zmk.unibe.ch (A.S.); 2 Department of Oral and Maxillofacial Surgery, Copenhagen University Hospital (Rigshospitalet), 2100 Copenhagen, Denmark; 3 Departamento de Periodontología, Universitat Internacional de Catalunya, 08195 Barcelona, Spain; amonjec@umich.edu

**Keywords:** peri-implantitis, biological complications, bone regeneration, dental implants

## Abstract

Dental implants may be considered a reliable routine procedure in clinical practice for the replacement of missing teeth. Results from long-term studies indicate that implant-supported dental prostheses constitute a predictable treatment method for the management of fully and partially edentulous patients. Implants and their restorations, however, are not free from biological complications. In fact, peri-implantitis, defined as progressive bone loss associated to clinical inflammation, is not a rare finding nowadays. This constitutes a concern for clinicians and patients given the negative impact on the quality of life and the sequelae originated by peri-implantitis lesions. The purpose of this narrative review is to report on the prevalence of peri-implantitis and to overview the indications, contraindications, complexity, predictability and effectiveness of the different surgical therapeutic modalities to manage this disorder.

## 1. Introduction

At the 2017 World Workshop on the Classification of Periodontal and Peri-implant Diseases and Conditions, new disease and case definitions were presented for peri-implant health, peri-implant mucositis and peri-implantitis [1].

Epidemiologic reports on peri-implant diseases across the globe demonstrated a wide range according to the population screened and the case definition adopted. Other factors, such as compliance with supportive therapy and characteristics of patient samples and implant recipient sites may influence heterogeneity in reporting and make comparisons among studies challenging. For example, outcomes of a publication on the effectiveness of implant therapy in a Swedish population sample indicated that significantly higher odds ratios for moderate/severe peri-implantitis were found for patients diagnosed with periodontitis (OR 4.08) compared with periodontally healthy patients [2].

Without discriminating between implants placed in native vs. augmented bone, the prevalence of peri-implant diseases was reported from longitudinal [3] and from cross-sectional studies, respectively [4,5,6,7,8,9,10]. The outcomes of a systematic review with meta-analysis reported a weighted mean prevalence of peri-implant mucositis of 43% (range: 19–65%) and of peri-implantitis of 22% (range: 1–47%), respectively [3].

Alveolar ridge augmentation is a frequent procedure in implant dentistry. Outcomes of clinical studies on the long-term survival rates of implants placed in augmented vs. pristine bone is still controversial. While some studies indicated comparable outcomes in terms of implant survival rates and marginal bone loss [11,12,13,14,15], other studies reported inferior results for implants placed in augmented sites [5,16,17,18].

Considering the difficulties in performing clinical trials with appropriate design and patient selection, the question of potential differences between the progression of peri-implantitis in augmented vs. native sites was addressed in a pre-clinical in vivo study using the experimental peri-implantitis model in Labrador dogs. The outcomes of that study indicated that the size and vertical dimension of the peri-implantitis lesion were larger at augmented sites compared with native sites. Moreover, it was demonstrated that implants with non-modified (i.e., turned) surfaces exhibited smaller amounts of bone loss and smaller dimensions of peri-implantitis lesions compared with implants with modified (i.e., micro-rough) surfaces [19].

It was the aim of the present narrative review to evaluate existing evidence on the prevalence and treatment outcomes of peri-implantitis focusing on surgical treatment protocols.

## 2. Prevalence of Peri-Implantitis

Although over the last decades dental implants proved to be highly effective in replacing teeth with survival rates exceeding 95% over 10 years [20], biological complications compromise implant longevity. Accordingly, there is an increase in the treatment needs to arrest such disorders. Before comparing biological complications and outcomes of implants placed in native vs. augmented bone, the prevalence of peri-implantitis and the caveats of its interpretation are presented below.

Firstly, described in 1993, at the First European Workshop on Periodontology, peri-implant diseases have been widely investigated. Outcomes from experimental mucositis and peri-implantitis studies highlighted the cause-and-effect relationship between bacterial biofilms accumulation and inflammatory tissue alterations [21,22,23] and a plethora of studies assessed clinical and histopathological features, disease definitions, prevalence, risk indicators and treatment modalities. Over time, numerous disease definitions have been proposed and different clinical parameters have been defined. Consequently, a wide range of prevalence have been reported and their results published in a systematic review with meta-analysis [3]. Moreover, in a case series of 86 patients with a very long follow-up (i.e., range of 21–26 years), peri-implant mucositis and peri-implantitis prevalence amounted to 54.7% and 22.1%, respectively [24].

It should be highlighted that adopting different cut-off thresholds of marginal bone loss, a wide range of prevalence of peri-implantitis was reported [3]. Defining a threshold of marginal bone loss >5 mm yielded a prevalence of peri-implantitis of 1% [25] whereas a threshold of 0.4 mm marginal bone loss increased the prevalence of peri-implantitis to 47% [26].

In addition, it has to be differentiated between cases with baseline radiographs in which 1–1.5 mm of marginal bone loss has been proposed to define peri-implantitis and cases without baseline radiographs in which 2 mm of marginal bone loss after the initial remodeling phase account for the definition of peri-implantitis [27].

When peri-implantitis prevalence is reported, the level of reporting (i.e., implant vs. patient) must also be taken into consideration. For example, the prevalence of peri-implantitis was reported to be 1% at patient— and 0.4% at implant-level, respectively [25].

When comparing the prevalence of peri-implant diseases and failure rates of implants placed in native versus augmented bone, studies are scarce although tissue deficiencies resulting from bone remodeling, trauma or infections do often occur. For example, following atraumatic tooth extraction buccal alveolar fracture was observed in 9%, bony dehiscence in 28% and complete buccal plate loss in 4%, respectively [28]. Consequently, augmentation procedures simultaneous or delayed to implant placement are commonly performed. Now, the question falls on whether or not differences in the prevalence of peri-implant diseases at implants placed in augmented vs. native sites exist. A systematic review including 8 studies addressed this issue [29]. After a follow-up of at least 10 years, the patient-based weighted mean prevalence of peri-implant mucositis amounted to 22.4% (95% CI: 6–38%) in native and to 19.6% (95% CI: 0–40%) in augmented sites, respectively. Although no statistically significant differences were observed with respect to the patient-based prevalence of peri-implantitis of implants placed in native (10.3%; 95% CI: 4–17%) vs. augmented sites (17.8%; 95% CI: 0–37%), patients treated with implants in augmented sites displayed higher variability in terms of peri-implantitis compared to patients with implants in native sites. Patient-based implant failures were observed in 2.5% of native and in 3.6% of augmented sites, respectively. Again, the studies included in that systematic review displayed a wide range of definitions of peri-implantitis [29]. For example, Roccuzzo and co-workers defined marginal bone loss of ≥2 mm as peri-implantitis [30] while Tenenbaum and co-workers set the threshold for bone loss at ≥4.5 mm [31].

**Figure 1:**
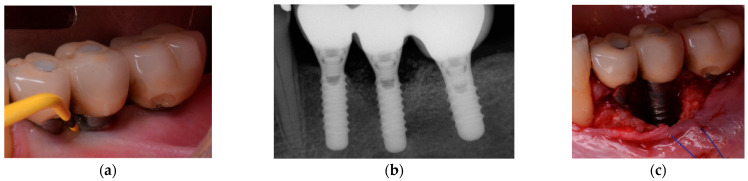
(**a**) Mandibular left premolar implant showing an increase in probing pocket depth as compared with previous records, bleeding and pus exhibiting shortly after probing. Note, the shallow vestibulum at the buccal aspect of the infected implant; (**b**) Radiographic image revealing significant bone loss. Note, the remaining particles of an anorganic bovine bone previously used for grafting; (**c**) Intra-operative appearance of the peri-implant infra-osseous defect after debridement. Note, the remaining particles of an anorganic bovine bone previously used for grafting simultaneously at implant placement stage.

**Figure 2:**
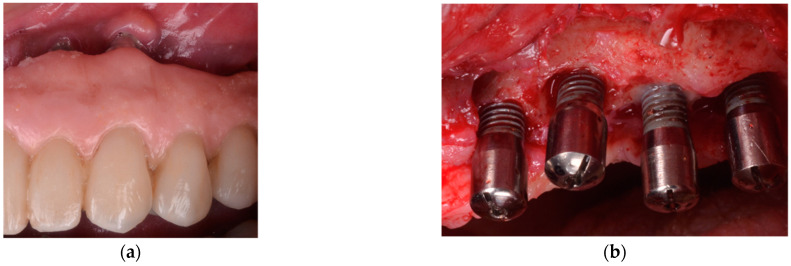
(**a**) Clinical appearance of implants placed in bone augmented with anorganic bovine bone and autogenous bone 5 years after placement in a smoker patient. Note, the poor plaque control and the inadequate prosthesis design that precluded adequate self-performed oral hygiene measures; (**b**) Intra-operative appearance of the peri-implant defects after debridement. Note, the predominant horizontal pattern of bone resorption.

**Figure 3:**
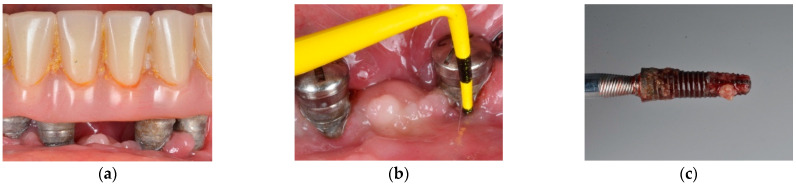
(**a**) Clinical appearance at several implant-supported fixed prosthesis involved affected of advanced peri-implantitis; (**b**) clinical probing indicates advanced attachment loss; (**c**) implant removal is suggested in advanced forms of peri-implantitis.

Taken together, studies on the prevalence of peri-implantitis yield a high heterogeneity in terms of case definitions, patient sampling and clinical scenarios (Figure 1, Figure 2 and Figure 3).

## 3. Surgical Treatment Approaches and Outcomes of Peri-Implantitis Therapy

Despite attempts to successfully treat peri-implantitis with non-surgical approaches (i.e., mechanical debridement) often in association with adjuncts (local and systemic antimicrobials, lasers, photodynamic therapy, etc.), it has been clinicians’ experience that the overall benefit in terms of changes in clinical parameters such as pocket probing depth (PPD) and bleeding on probing (BoP) is limited [32]. Indeed, due to the frequent presence of deep peri-implant pockets, different macro and micro implant surface characteristics, and difficult access for biofilm removal, access to implant surfaces for decontamination may be extremely challenging. Therefore, the use of nonsurgical protocols should be performed with the aim of preparing healthier peri-implant soft tissue conditions prior to adjunctive surgical therapy [32,33].

Following a treatment sequence taken from the periodontal surgical literature, the surgical management of peri-implantitis initiates with the elevation of a full-thickness flap to get access to the contaminated implant surface. Consequently, after peri-implant soft-tissue defect degranulation, an accurate surface decontamination should be performed. Over the years, several hand (i.e., titanium and teflon curettes) and power-driven instruments have been developed (i.e., glycine powder, ultrasonics, titanium and chitosan brushes) with the aim to optimize biofilm removal without altering the implant surface. Nonetheless, at the present time, none of the investigated tools have been proven to be superior in terms of peri-implant surface decontamination [34]. Therefore, it is suggested to combine a mechanical and chemical decontamination process prior to the assessment of the peri-implant bone defect configuration.

Following implant surface decontamination, four main surgical modalities have been described in the literature for treating peri-implant bony defects.

### 3.1. Open Flap Debridement without Resective Procedures

Open flap debridement (OFD) includes elevation of a mucoperiosteal flap with additional removal of granulation tissue in order to gain access to the contaminated implant surface. Following removal of the inflammatory tissue, the implant surface is decontaminated by means of mechanical, chemical, and/or additional (e.g., photodynamic therapy and laser) methods followed by mucoperiosteal flap repositioning and closure. Studies evaluating the outcomes of open flap debridement without resection procedures have reported high survival rates and moderate composite success rates (e.g., PPD ≤ 5 mm, absence of bleeding/suppuration on probing, and absence of progressive bone loss) up to 5 years following treatment [35,36]. More specifically, the results obtained by Heitz-Mayfield and co-workers after OFD with adjunctive delivery of systemic antimicrobials (i.e., amoxicillin 500 mg and metronidazole 400 mg, 3x/day for 7 days) reported complete disease resolution of around 19 (53%) implants in 15 (63%) patients. These results underlined the challenges for maintaining mid- to long-term, the positive short-term results.

### 3.2. Open Flap Debridement with Resective Procedures

Outcomes of a five-year study evaluating a resection approach with bone recontouring and adjunctive delivery of systemic antimicrobials indicated that 54% of implants yielded a successful outcome defined as disease resolution [37]. Indeed, 57 implants (44%) displayed disease recurrence/progression and among these, 27 had to be removed. Interestingly, a statistically significant correlation between residual PPD ≥ 6 mm (OR 7.4, 95% CI 2.8–19.3) and reduced marginal bone levels (OR 1.4, 95% CI 1.1–1.7) at the one-year follow-up and recurrence/progression of peri-implantitis was found. Moreover, implants with a modified surface were found to be at higher risk for disease progression as compared with implants with a non-modified surface (OR 5.1, 95% CI 1.6–16.5).

Comparable results were reported by Berglundh and co-workers in a long-term retrospective cohort study with an observation period of up to 11 years (2–11 years) [38]. All the 95 implants placed in 50 patients underwent OFD followed by osseous recontouring to obtain pocket elimination. The clinical and radiographic results revealed positive outcomes in terms of PPD and BoP changes and confirmed how implant surface characteristics (i.e., turned vs. modified surfaces) have an impact on the final outcomes [38]. The impact of the implant surface characteristics on implant survival and success rates after surgical treatment of peri-implantitis has also been assessed by other authors [39,40]. Indeed, at the seven- and 10-year follow-up adopting a reconstructive surgical protocol using a deproteinized bovine bone mineral (DBBM) with 10% collagen, patients rehabilitated with sand-blasted and acid-etched (SLA) implants presented an implant survival rate of 80%, while patients who received implants with a titanium plasma sprayed (TPS) surface presented an overall survival rate of 55%.These results indicated that implants with a moderately rough surface (i.e., SLA) performed better as compared with those with a higher surface roughness (i.e., TPS).

Comparable results were recently reported in a long-term retrospective study with a follow-up of 11 years [41]. All the implants which had to be removed before the final examination had a modified surface (*n* = 8, 14%), while implants with a turned surface did not experience any loss [41].

**Figure 4:**
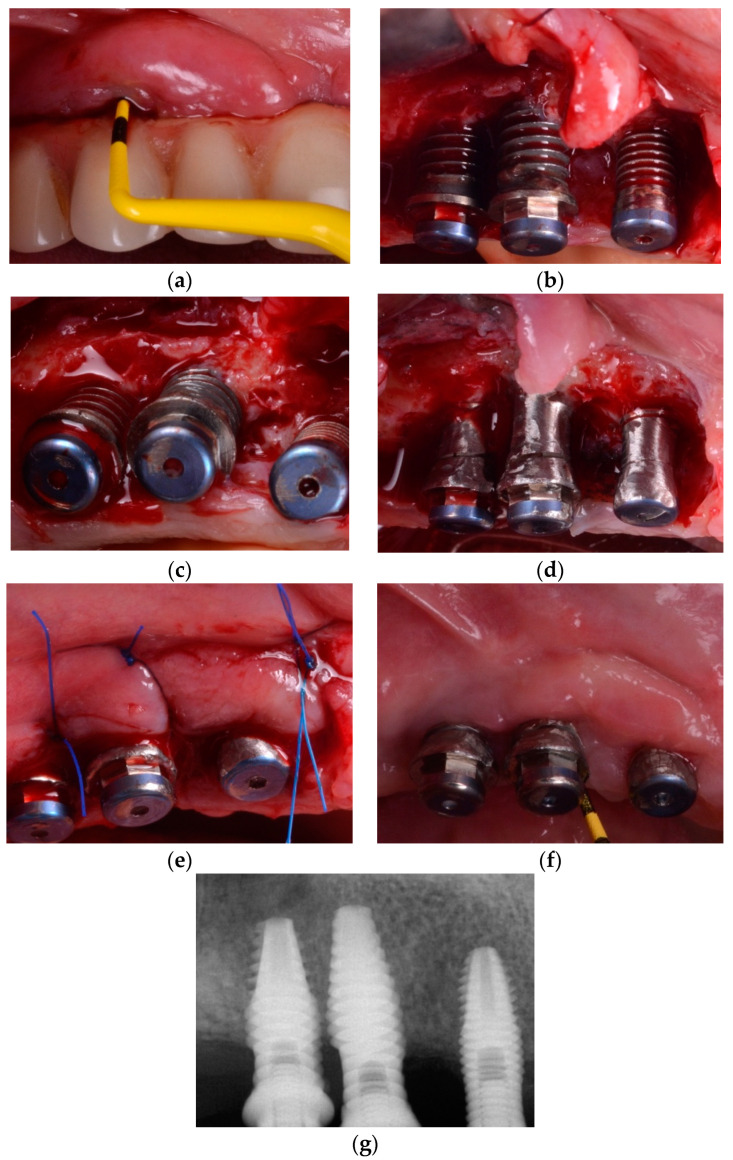
(**a**) Clinical presentation of peri-implantitis; (**b**) Access flap reveals moderate bone loss (<50%) (frontal view). Note the supra-crestal defect morphology; (**c**) Occlusal view of moderate bone loss; (**d**) Implantoplasty was performed as adjunct to the surgical resective therapy of peri-implantitis (frontal view); (**e**) Occlusal view of the implantoplasty and bone topography after osteoplasty to reach a flat bone architecture; (**f**) clinical resolution of peri-implantitis at 6-month follow-up; (**g**) bone stability is noted upon radiographic assessment.

Implantoplasty, which is defined as removal of the supracrestal implant threads and exposed surface, can also be performed as part of a resection approach. The rationale for performing an implantoplasty procedure is to alter the implant surface topography in order to facilitate biofilm removal by the patient. Controversial evidence, however, exists in terms of the advantages of implantoplasty procedures as compared with other methods of decontamination in the surgical management of peri-implantitis (Figure 4).

In the surgical management of peri-implantitis, the efficacy of implantoplasty was investigated and compared with a bone resection approach alone in terms of marginal bone level (MBL) changes over a follow-up period of 3 years [42]. In that study, 10 patients were treated with open flap debridement (OFD) in combination with implantoplasty, while nine patients underwent a bone recontouring procedure alone. At the three-year evaluation, peri-implant MBL changes following implantoplasty were significantly smaller as compared with those observed in the control group.

Results of a case series study indicated that a combined bone resection procedure with implantoplasty resulted in marginal bone level stability and disease resolution in 89% of the implants after a mean follow-up of 3.4 years [43].

Recently, the efficacy of implantoplasty was evaluated and compared with that obtained using an air-polishing device with glycine powder in the surgical management of peri-implantitis in a six-month randomized clinical trial [44]. Following flap elevation and removal of granulation tissue, implant surfaces were either decontaminated with implantoplasty (*n* = 22) or with the air-polishing device (*n* = 20) and no grafting materials or barrier membranes were applied within the defects. At the three- and six-month clinical examinations, comparable clinical results in terms of changes in PPD and BoP were observed between the two groups indicating that implantoplasty was as effective as glycine air polishing in the surgical management of peri-implantitis [44].

Unfortunately, there is lack of clinical evidence describing the consequences of implantoplasty procedures such as implant fracture and presence of titanium particles in the peri-implant soft tissues [45]. Therefore, the question whether a procedure such as implantoplasty should be recommended or not to decontaminate implant surfaces remains controversial.

### 3.3. Reconstructive Procedures

**Figure 5:**
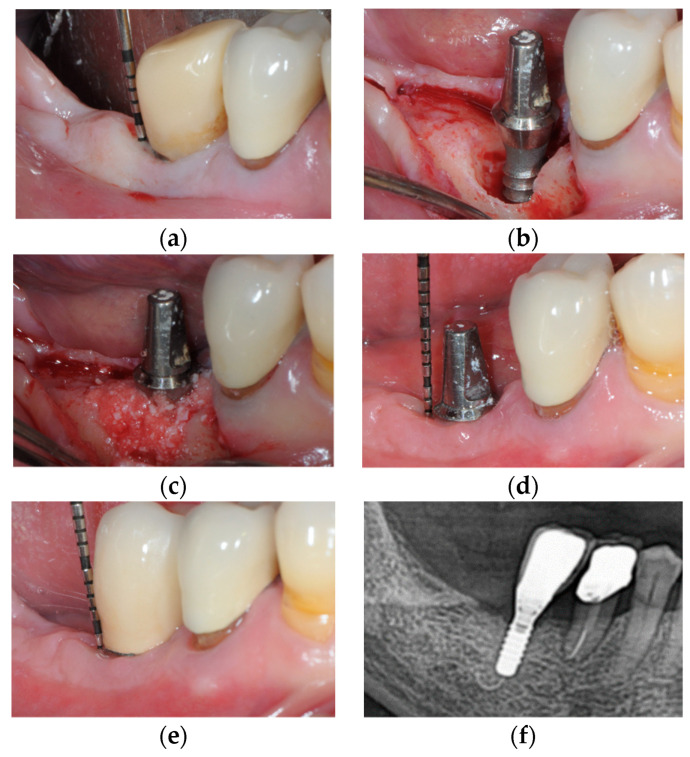
(**a**) Mandibular right premolar implant installed in pristine bone showing increased probing pocket depth as compared with previous records, bleeding and pus exhibiting shortly after probing; (**b**) Intra-operative appearance of the peri-implant infra-osseous defect after debridement; (**c**) Anorganic bovine bone mineral with 10% collagen is applied in the infra-osseous component; (**d**) Six-month follow-up after non-submerged healing, no signs of inflammation and peri-implant probing depth was noted to be consistent with health; (**e**) One-year follow-up after delivery of the final restoration. Note, peri-implant stability; (**f**) Radiographic image, at 1-year follow-up, reveals substantial bone fill.

Following preclinical [46] and clinical [47] evidence of the possibility to obtain re-osseointegration around dental implants affected by peri-implantitis, several studies proposed different reconstructive protocols using autogenous bone and/or various bone substitutes (Figure 5) and barrier membranes to treat peri-implantitis defects [48,49,50,51,52,53,54,55,56].

A randomized controlled trial (RCT) evaluated the adjunctive use of a demineralized bovine bone substitute and failed to report additional radiographic hard tissue fill as compared with open flap debridement alone [56]. However, a composite assessment of clinical and radiographic parameters identified that surgical debridement with adjunctive use of a bone substitute was more predictable as compared with open flap debridement alone in contained defects with 3–4 bony walls [56].

Results of a recent systematic review [57] reported limited evidence available from three RCTs [58,59,60] with a total of 116 implants evaluating the effects of a reconstructive approach as compared with open flap debridement alone and indicated a benefit in radiographic marginal hard tissue level changes when using a reconstructive approach. However, the reconstructive protocols described in the three RCTs included in the systematic review (i.e., adjunctive use of porous titanium granules and enamel matrix derivative) do not represent commonly used surgical procedures to treat peri-implantitis defects. In addition, the porous titanium granules are no longer commercially available.

In terms of disease resolution, no significant differences in clinical parameters (i.e., reduction in PPD and BoP scores) were observed as compared with a reconstructive approach with open flap debridement alone [57].

### 3.4. Combined Resective and Reconstructive Procedures

The use of a combined resection (i.e., implantoplasty of the supracrestal portion of the implant) and reconstructive (i.e., filling of the infrabony component of the defect) approach has been described adopting transmucosal [61,62] and submerged [63] healing protocols, respectively.

Outcomes of a combined surgical protocol including implantoplasty of the suprabony component and application of a DBBM grafting material with a collagen membrane in the intrabony portion of the peri-implant defect were reported in a 12-month case series study. Statistically significant clinical improvements in terms of PPD and Clinical Attachment Level (CAL) changes and an increase in mucosal recession were observed after 1 year [61].

Changes in clinical parameters of implants treated with a combined resection (i.e., implantoplasty of the supracrestal implant portion) and reconstructive approach were reported in a study including 15 patients with a follow-up of 7 years [62]. Following surface decontamination with either an Er:YAG laser or plastic curettes with cotton pellets and saline solution, the intrabony components of the peri-implant defects were treated by means of a grafting material and a collagen membrane. Comparable clinical conditions in terms of BoP reduction and CAL gain were reported in both groups irrespective of the method of surface decontamination [62].

The clinical and radiographic results in the reconstructive management of two- or three-wall peri-implantitis defects were reported in a case series study [63]. Fifteen patients diagnosed with peri-implantitis underwent a combined resection (i.e., implantoplasty of the supracrestal implant portion) and reconstructive (i.e., filling of the intraosseous compartment) approach followed by a submerged healing without restorations in situ for 8–10 weeks. At the 6- and 12-month follow-up examinations, statistically significant reductions in PPD and BoP, as well as radiographic hard tissue fill, were recorded. Owing to the marked mean post-surgical mucosal recession of 2.5 mm, a treatment which has been proven to be of limited efficacy [64], this combined surgical approach may be applied to posterior implant areas of limited esthetic priority [63].

### 3.5. Soft Tissue Conditioning with Resective Procedures

The significance of keratinized mucosa on tissue health has been demonstrated [65,66]. On the contrary, the influence of keratinized mucosa on the therapeutic outcomes of peri-implantitis remains unclear [67].

**Figure 6:**
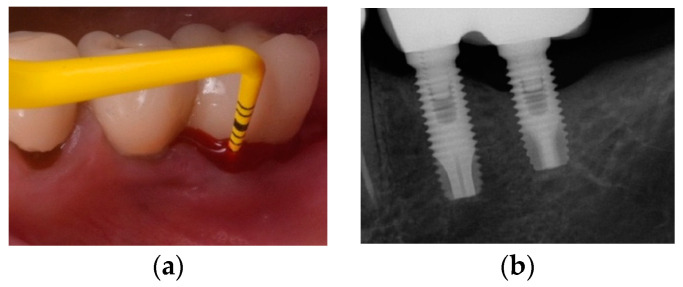

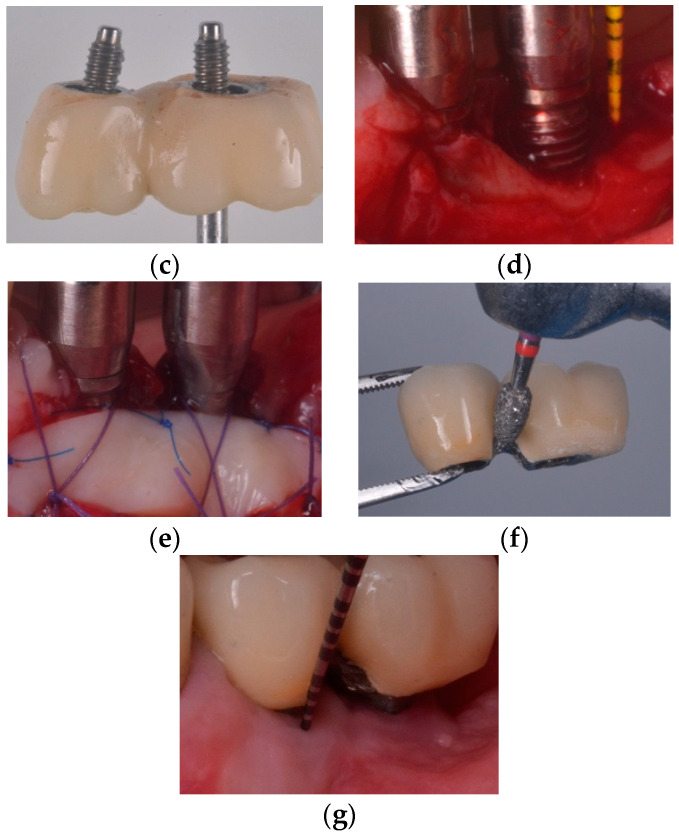
(**a**) Clinical presentation of peri-implantitis; (**b**) Radiographic image compatible showing moderate (<50%) bone loss; (**c**) Inadequate prosthesis emergence profile; (**d**) Partial-thickness apical position flap; (**e**) Soft tissue conditioning by means of free epithelial graft; (**f**) Prosthesis contour modification to facilitate proximal access during self-performed oral hygiene; (**g**) Clinical resolution of peri-implantitis associated with a gain of keratinized mucosa.

Recently, a protocol has been described to manage supra-crestal and/or dehiscence-type peri-implantitis bone defects associated with lack of keratinized mucosa (Figure 6). Four steps define the success of this: (1) partial-thickness flap apically positioned, (2) bone recontouring to achieve a flat architecture, (3) implantoplasty for the exposed implant surface and, (4) free epithelial graft stabilized on the vascular recipient bed. A prospective case series [68] demonstrated complete disease resolution in 78.6% of the patients and 87.1% of the peri-implantitis implants. Interestingly, unsuccessful cases were associated with less gain of keratinized mucosa, deep pocket probing depths, bleeding on probing, and less satisfaction during brushing at 12 months.

## 4. Conclusions

The following conclusions can be drawn from the present review:

- Clinical and radiographic features define peri-implantitis
- Prevalence of peri-implantitis relies upon case definition.
- Patient’s characteristics such as history of periodontitis, tobacco consumption, compliance with supportive therapy as well as implant topographic characteristics differed considerably among studies, limiting thus, the interpretation and generalizability of the obtained outcomes.
- Based on the limited effectiveness of non-surgical procedures, a wide range of surgical protocols and biomaterials used for the management of peri-implantitis lesions have been described in the literature with scarce long-term evidence.
